# Successes and Challenges on the Road to Cure Hepatitis C

**DOI:** 10.1371/journal.ppat.1004854

**Published:** 2015-06-18

**Authors:** Stacy M. Horner, Susanna Naggie

**Affiliations:** 1 Department of Molecular Genetics and Microbiology, Duke University Medical Center, Durham, North Carolina, United States of America; 2 Department of Medicine, Duke University Medical Center, Durham, North Carolina, United States of America; 3 Duke Clinical Research Institute, Durham, North Carolina, United States of America; University of Florida, UNITED STATES

## Introduction

Hepatitis C virus (HCV) represents a significant health burden worldwide, with an estimated 185 million people chronically infected [1]. A leading cause of liver transplantation, HCV infection can result in severe liver disease including cirrhosis and hepatocellular carcinoma [2]. Cure of HCV infection results in substantial decreases in such liver-related morbidity and mortality [3]. Prior therapies for HCV offered only 40% cure for the most difficult-to-treat genotype-1 infection, required 48 weeks of therapy with an injectable interferon, and included significant adverse events [4]. The past year has seen the approval of five interferon-free direct-acting antiviral (DAA) regimens for HCV, including combinations of DAAs and fixed-dose combination pills (Tables 1 and 2) [5–13]. Sustained virologic response (SVR), the virologic surrogate for clinical cure, has improved to >90% for most populations across all HCV genotypes (Table 2). While the successes attributable to DAA combination therapies will be many, there also remain challenges and much for us to learn as we embark on this journey to eradicate HCV. Here, we will discuss several of the greatest successes and future challenges in HCV therapeutics today.

**Table 1:** All-oral direct acting antiviral regimens available for clinical use.

Regimen	DAA Mechanism of Action	Approved Genotype Coverage
**Daclatasvir + Asunaprevir** (24 weeks regardless of cirrhosis or prior treatment experience) [5,6]	NS5A inhibitor + NS3–4A protease inhibitor	1b
**Daclatasvir + sofosbuvir ± ribavirin** (12 weeks for GT1 and GT4 in patients without cirrhosis, 24 weeks for GT1 and GT4 with compensated cirrhosis, 24 weeks for GT3 with prior treatment experience or cirrhosis) [7–9]	NS5A inhibitor + NS5B nucleotide inhibitor	1–4
**Ledipasvir/sofosbuvir** (12 weeks except in treatment-experienced patients with cirrhosis, who receive either 12 weeks with ribavirin or 24 weeks* without) [10]	NS5A inhibitor/NS5B nucleotide inhibitor	1
**Paritaprevir/ritonavir/ombitasvir + dasabuvir ± ribavirin** (12 weeks except in all GT1a-infected patients with cirrhosis; all GT1a patients receive ribavirin, and only GT1b patients with cirrhosis receive ribavirin) [11]	NS3–4A protease inhibitor/NS5A inhibitor + NS5B nonnucleoside inhibitor	1
**Simeprevir + sofosbuvir** (12 weeks except in all patients with cirrhosis, who receive 24 weeks*) [12]	NS3–4A protease inhibitor + NS5B nucleotide inhibitor	1
**Sofosbuvir + ribavirin** (12 weeks for GT2 infection, 24 weeks* for GT1 and GT3 infection) [13]	NS5B nucleotide inhibitor	1–3

*24 weeks of treatment; DAA, direct-acting antiviral; GT1 (a/b), genotype-1; GT2, genotype-2; GT3, genotype-3

**Table 2:** Sustained virologic response for all-oral direct acting antiviral regimens.

Regimen		Treatment Naïve	Treatment Experienced	Treatment-Naïve Cirrhosis	Treatment-Experienced Cirrhosis
**Daclatasvir + Asunaprevir**	Japan GT1b	87%+	81%	N/A	N/A
	Global GT1b	90%	82%		
**Daclatasvir + sofosbuvir ± ribavirin**	GT1a	96%	97%	-	-
	GT1b	100%	100%	-	-
	GT2	92%*	-	-	-
	GT3	90%	86%	58%	69%
**Ledipasvir/sofosbuvir**	GT1	-	-	94%	100%
	GT1a	98%	95%	-	-
	GT1b	100%	87%	-	-
**Paritaprevir/ritonavir/ombitasvir + dasabuvir ± ribavirin**	GT1	-	-	94%*	92%*
	GT1a	97%	96%	-	-
	GT1b	100%	100%	-	-
**Simeprevir + sofosbuvir**	GT1	94%	-	100%*	93%*
	GT1a	-	89%	-	-
	GT1b	-	94%	-	-
**Sofosbuvir + ribavirin**	GT1	-	-	60%*	-
	GT1a	82%*	-	-	-
	GT1b	54%*	-	-	-
	GT2	95%	86%	86%	72%
	GT3	93%*	77%*	92%*	60%*

^+^Interferon ineligible/intolerant;

*24 weeks of treatment; GT1 (a/b), genotype-1; GT2, genotype-2; GT3, genotype-3; N/A, not available

## Potent DAA Combinations Are Closing the Gap for Unique Patient Populations

Although all patients with HCV infection will benefit from the availability of oral DAA combination therapies, several unique patient populations have seen dramatic improvements in SVR, revealing the great clinical impact of these therapies. Specifically, patients with HIV/HCV dual infection, patients with compensated and decompensated cirrhosis, and patients who have had liver transplantation for HCV-associated liver disease are exhibiting fantastic (87%–98%) SVR rates with oral DAA combination therapy [14–16]. These patients, who have a greater risk of progression of liver disease, hepatocellular carcinoma, and death, previously had low response rates or even absolute contraindications to treatments with interferon [17–18].

The potential impact of HCV eradication for these highest-risk populations could be enormous, including decreases in hepatocellular carcinoma and liver transplantation, as well as prolonged survival for those already living with liver grafts. And yet, we do not know for sure how this story ends. While the liver has the ability to repair injured tissue [19] and there is evidence to suggest that fibrosis induced by chronic HCV infection is reversible [3], is there a point of no return? Previously, patients with HCV-associated decompensated liver disease (ascites, hepatic encephalopathy, and variceal bleeding) had only one choice for survival: organ transplantation. Is viral eradication enough to change their fate? The largest study to date in patients with decompensated cirrhosis reported SVR of nearly 90% with a DAA combination regimen [15]. Importantly, the study also reported on improvement in serum markers of synthetic liver function for a majority (>50%) of patients at post-treatment week 4. Whether these improvements translate to improved clinical outcomes over time remains to be seen, but there is reason for optimism.

## HCV Genotype Diversity Has Complicated the Search for a Pangenotypic Regimen

**Fig 1:**
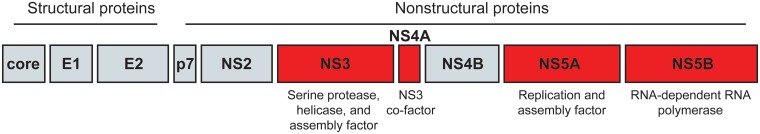
The HCV proteins. The HCV polyprotein is processed into the structural and nonstructural proteins of the virus, as shown here. The NS3–4A, NS5A, and NS5B proteins, all targets of newly developed direct-acting antivirals for HCV, are highlighted in red and their major functions described.

HCV is a positive-stranded RNA virus. Its 9.6-kb genome is translated into a polyprotein that is processed into structural and nonstructural (NS; including NS3, NS4A, NS4B, NS5A, and NS5B) proteins (Fig 1) [20]. The NS proteins are the targets for the current approved DAA, including NS3–4A protease inhibitors (PI), NS5A inhibitors, and NS5B nucleot(s)ide (NA) and non-nucleoside (NNA) analogues (Table 3). A major challenge to the design and implementation of DAA for HCV is the incredible genetic diversity of HCV. HCV contains six major genotypes, as defined by phylogenetic and sequence analysis of the viral genome. These genotypes vary by 30%–35% at the nucleotide level and contain nearly 70 subtypes [20]. Clinically, this genetic diversity has translated into different regimens based on genotype and even subtype (1a versus 1b).

**Table 3:** Properties of HCV direct-acting antivirals.

Direct-Acting Antivirals	Drug Type	Mechanism of Action	Genotype Coverage	Efficacy	Barrier to Resistance	Examples
**NS3–4A Inhibitors**	Peptidomimetic compound	Inhibits NS3 protease active site to prevent HCV polyprotein cleavage	Narrow to medium (in first-generation drugs)	High	Low	Asunaprevir, paritaprevir, and simeprevir
**NS5B Polymerase Inhibitors**	Nucleotide analogue	Binds to highly conserved active site of NS5B	Broad	Medium to high	High	Sofosbuvir
**NS5B Polymerase Inhibitors**	Non-nucleoside analogue	Allosteric regulator of NS5B	Narrow	Low to medium	Low	Dasabuvir
**NS5A Inhibitors**	Small molecular compound	Binds to domain I of NS5A, inhibits replication and assembly	Medium to broad	High	Low	Daclatasvir, ledipasvir, and ombitasvir

The ideal HCV DAA regimen would have pangenotype efficacy. However, because of the viral genetic diversity and mechanisms of action of the DAAs, this has been difficult to achieve. Of the five interferon-free DAA regimens available in either the United States or Europe, only two have been approved as pangenotypic regimens. The regimen of sofosbuvir, a first-in-class NA, and ribavirin was approved for genotypes 1–4 and has in vivo evidence to support efficacy in genotypes 5 and 6 [13]. While this regimen remains the standard of care for genotypes 2 and 3, its efficacy was suboptimal in the most common HCV genotype in the US, genotype 1. Daclatasvir, a pangenotypic NS5A inhibitor, when combined with sofosbuvir, provides the most potent pangenotypic activity to date and was approved in Europe for genotypes 1–4. Daclatasvir has good activity against genotype 3 and maintains activity against genotype 2 polymorphisms [7]. Unfortunately, the recently reported efficacy of 12 weeks of this regimen in genotype 3 cirrhotic patients was quite poor [8]. Other DAA regimens either lack pangenotypic coverage or have pangenotypic activity but lack clinical data in all genotypes. Ledipasvir, an NS5A inhibitor approved in combination with sofosbuvir, loses activity for a majority of genotype 2 infections because of a common NS5A polymorphism and has suboptimal in vitro activity versus genotype 3 [10]. Simeprevir, a first-generation PI approved in combination with sofosbuvir, has limited activity versus genotype 3 and has not been studied in humans for genotypes 2, 5, or 6, although it has in vitro activity [21]. The most recently approved regimen, which combines three DAAs (all with low barrier to resistance): a pangenotypic NS5A inhibitor, ombitasvir, used with a first-generation PI (paritaprevir) and an NNA (dasabuvir), is limited by the narrow genotype 1 activity of the NNA [11].

This fragmentation of treatment by genotype complicates the clinical approach to care and limits the feasibility of HCV treatment in the resource-limited setting, where genotyping and access to multiple regimens is not feasible. Moving forward, investigational second-generation PIs with broad genotypic coverage (such as MK-5172), highly potent pangenotypic NS5A inhibitors (including GS-5816), and triple DAA combinations with NA backbones are expected to help us achieve more potent pangenotypic coverage, including better options to treat genotype 3.

## The Clinical Role of Resistance-Associated Variants (RAVs) Is Becoming More Clear

Viral sequences with preexisting polymorphisms can present a therapeutic challenge. The best clinical example is the preexisting NS3 Q80K polymorphism, found in 5%–48% of those with genotype 1a. This polymorphism confers resistance to simeprevir and limits its efficacy (SVR 58% versus 84%; in combination with pegylated interferon and ribavirin), thereby necessitating pretreatment polymorphism testing [12]. Combining simeprevir with the potent DAA sofosbuvir appears to overcome this limitation only in patients without cirrhosis [22,23]. Nucleotide analogues including sofosbuvir have exceptional genetic barriers to resistance. The signature RAV with sofosbuvir is the S282T mutation, which was not detected in any patient at baseline or time of virologic failure in the sofosbuvir phase 3 program [24]. Other treatment-emergent variants (TEVs) have been reported in the phase 3 program, including the L159F and V321A mutations. The change in sofosbuvir EC50 for these TEVs does not appear to be clinically significant, and retreatment of subjects with these TEVs with sofosbuvir-containing regimens did not support a role for them in treatment failure. Baseline NS5A polymorphisms that confer resistance are significantly more common and will likely be more problematic in the setting of retreatment. In a pooled resistance analysis of the ledipasvir/sofosbuvir phase 3 programs, while only 16% of patients had NS5A RAVs at baseline, significantly more patients (43%) suffering virologic failure harbored these RAVs at baseline [25]. There are now several patient subgroups in which baseline NS5A RAVs may lower SVR, including genotype 3 patients with cirrhosis treated with daclatasvir and sofosbuvir and treatment-experienced patients receiving ledipasvir and sofosbuvir [25,26]. While baseline polymorphism testing is not recommended at this time because of the overall high SVR (>90%), it may become more relevant in the setting of retreatment. The first retreatment study of prior NS5A failures confirmed that evidence of NS5A RAVs at time of retreatment confers greater risk of relapse [27].

The ability of HCV to develop de novo resistance to antiviral drugs is quite high. HCV replicates as a quasispecies; therefore, RAVs can preexist within the viral population at baseline and emerge as the dominant species during treatment. The high mutability of HCV has to do with the high error-prone nature of the HCV RNA-dependent RNA polymerase and large viral populations [28]. In fact, it has been predicted that in a single day, HCV can generate genomes with all possible single and double nucleotide changes, and as long as these genomes maintain fitness, they could confer antiviral resistance [28]. This same model predicted that combination DAA regimens would require a genetic barrier of four or more resistance mutations to achieve clinical efficacy.

Each class of DAAs can select for RAVs; however, the genetic barriers and fitness of these RAVs varies. NAs inherently have high barriers to resistance, because they directly target the conserved polymerase active site and resistant variants have low fitness [28]. On the other hand, NS5A inhibitors, PIs, and NNAs all have low barriers to resistance, with single amino acid substitutions conferring high-level resistance. In the minority of patients who suffer virologic failure during DAA combination therapy, dual and triple RAVs are being reported [10–13].

What happens to RAVs after the cessation of treatment? This depends on the fitness of those variants. For first-generation PIs, a recent report of long-term follow-up of patients treated with boceprevir (first-generation NS3–NS4A PI) found that after 3 years, 27% of patients still had RAVs and that the median time for all RAVs to become undetectable was 1.11 years [29]. This carries important clinical implications for retreatment decisions in a patient with RAVs. For example, NS5A RAVs exhibit more replicative fitness and can persist for >96 weeks [30]. Early retreatment studies suggest this will impact success of retreatment and thus significantly limit options for patients with NS5A RAVs for the foreseeable future [27].

## Even with Highly Effective DAA Combination Therapies, Some Historic Baseline Predictors of SVR Remain

One of the greatest surprises thus far in the new era of treating HCV is that some of the same factors that predicted response to interferon therapies still play a role in DAA response rates. Although SVR rates are high across all subgroups, recent pooled multivariable analyses of phase 3 trials provide some granularity on treatment response. While these predictors may vary across regimens, higher rates of relapse are being reported in patients based on genotype subtype, presence of cirrhosis (in particular those with prior treatment failure), *IL28B* TT genotype, sex, race, and higher baseline HCV RNA [31,32]. Based on the presence or absence of these predictors, some patients may achieve SVR with 8 weeks of therapy, while others require 24 weeks. Moving forward, these “difficult-to-treat” populations should be targeted for innovative approaches to therapy so that no patient is left without a chance of cure.

## Our Greatest Challenge Just Might Be of Our Own Making

Direct-acting antivirals for HCV are likely to be heralded as one of medicine’s greatest advancements. The possibility of eradicating HCV from the globe seems within arm’s reach. With SVR pushing 100% for many HCV-infected populations and ongoing studies pushing the limits of treatment to just 6 weeks with triple DAA combinations, there is much reason for hope. Yet, the unfortunate reality is that because of the high cost of these medications, many patients will be denied coverage and the opportunity for cure. Many payers are only providing coverage to those patients with the highest stages of fibrosis. Is there another precedent in medicine in which we don’t treat a curable transmissible disease? The recent exclusivity agreements between Express Scripts and AbbVie and CVS/Caremark and Gilead mark a new era in the battle against HCV, although in this case we seem to be battling against ourselves. We should carefully consider the consequences of pricing and such relationships, as much of this has overshadowed the bright light of medical innovation—what a shame.
